# In Nature's School

**Published:** 1889-08-17

**Authors:** 


### The Doctor's Pulpit.

### IN NATURE'S SCHOOL.

### Trained or Strained.

Everything in nature has its measure and its limitation. To develop a living organism to its full potentiality, and at such a rate of progress as is natural to it, is reasonable and profitable. But to attempt to develop it beyond its natural possibilities is irrational, and usually ends in waste and destruction. Careful breeding, rearing, and training have given us horses of remarkable swiftness and staying power. But extraordinary as are the speed and endurance of the best English racehorses it is the easiest thing in the world to so ruin them by excessive training that they shall not only win no first-class races, but shall not even be fit for the ordinary work of the hack or the carriage-horse. The breeds of sheep have been improved in a high degree both in size and quality of wool. The flesh, however, of the smaller and older breeds is held by epicures to be very superior to that of the larger and more modern varieties; and this experience is so common that Scotch or Welsh mutton commands considerably higher prices in the market than Leicester or Southdown. Moreover, even in the case of the breeds that have been so developed by selection and careful rearing it is found that progress is not attainable beyond definite limits; that at certain stages the animals tend to become much less robust in health, and therefore of less constant value. Reversion, also, to the original type in respect of size and other characteristics becomes much more frequent in the case of the more highly-developed varieties. The same law holds absolutely in regard to cattle and pigs. There are limits beyond which Nature will not permit encouraged development or training to pass.

This is the day of science. On all subjects men are asking, "What does science say?" Science speaks for herself, and she also speaks by the mouths of her interpreters. To those who have no eyes and no patience she speaks by her prophets, who are called "scientific men" and "specialists;" but to those who have eyes and patience to use them she speaks with her own voice. It is not desirable to undervalue the utterances of scientific prophets on the ground that Nature opens her book freely and permits all men to read it for themselves if they will. The world can never be anything but grateful to its Aristotles, its Bacons, its Newtons, and its Faradays. Nevertheless, every man should strive to make himself master of at least one or two chapters in the book of natural science, because he will then be so much better able to understand all the other chapters, and to duly weigh and check the sayings and doings of those whose business it is to make known the ways of Nature to mankind.

The plain man, without the intervention of the specialist, has discovered that Nature has laws and limitations. But the plain man often fears to trust his native sense and proved experience. He thinks that science is something different from common knowledge and everyday work. But the most sure and reliable science of all is that which is known and proved by universal experience. The specialist generally knows more, and more accurately than the plain man; but that which the plain man does know, and knows by the sure test of repeated experience, is as good science, and as much to be relied upon, so far as it goes, as the more painfully elaborated conclusions of the specialist. The best sense of the ages, what Tennyson has called "the common sense of most," will be the final court of appeal to try the accuracy and the value of scientific discoveries and of medical progress, exactly as it has been the court of appeal which has decided the merits and the place in the world of Homer, of Shakespeare, and of St. Paul.

It does not require special knowledge to enable a man to understand that a racehorse may be "trained" or "strained"; that if he be judiciously trained he will at the right moment make the best use of his natural powers; and that if he be strained, "overstrained," as it is the custom to call it, he will probably break down just when the most serious consequences are depending upon his holding out. It is equally clear and evident that the same thing may happen to the man who runs a mile-race, to the "'Varsity oar," to the "coached" student who is reading for honours, to the zealous clergyman who cannot endure wrong-doing in his parish, to the clever boys in the board school who are to gain the highest attainable grants, or to the stupid ones who are to be prevented at all hazards from covering the school with disgrace.

The "trainers" in all these cases are too apt to become "strainers." They teach too little and they drive too much. That is the secret of the failure of thousands of boys in schools, of students at colleges, of clerks in offices, and of men in life. Foolish trainers think it is the fault of the trained. No such thing! It is Nature, outraged, defied, trampled upon, that rises up in stern rebellion, and compels the teacher and the master to see by their miserable failures how irrational and even wicked they have been. The man who thinks to drive his way through life and to gain his ends by mere force of will is but a fool in ultimate analysis, and is fore-doomed to failure from the very outset. Nature insists upon being duly regarded on all occasions, and she has ample means for compelling obedience. Neither the clever nor the stupid, the wilful nor the careless, the religious nor the profane need hope to defeat or evade her. She is always and everywhere a force to be reckoned with, and a force that can neither be persuaded, nor deceived, nor defied.

Is the present an age of "training" or of "straining"? It is an age of both. But the training constantly tends to degenerate into straining. Even the man who has passed beyond the care of "tutors and governors" is sometimes in such mad haste to reach the goal he is aiming at that he blinds his eyes and stops his ears to every consideration of reason and feasibility. Such a man often ends by smashing his head against a rock that stands in his way, or breaking his neck in a fall down a precipice which he might have seen a mile off if he would have run the race with his eyes open. The highest of life's prizes are not to the rash, but to the reasonable, not to the "strained," but to the "trained." "Training" is the very lifeblood of success. Next to natural capacity it is the one indispensable condition on which the winning of the race depends. Just as certain is it that "straining" is the madman's whip by which he drives his team until it can gallop no longer, and all the horses stand panting and trembling, or fall dead by the roadside. How strange it is that reason is so feeble in men whilst impulse and desire are so over mastering and uncontrollable! From the days of the cave-man until now, Reason, which is Nature, has added progress to progress and victory to victory. And yet Reason and Nature are the very last companions which the majority of mankind seek to be on terms of intimacy with. Happy is the man who clearly sees that whilst Nature is his stern guardian, she is also his unerring teacher and his faithful friend.

